# *Drosophila* D-idua Reduction Mimics Mucopolysaccharidosis Type I Disease-Related Phenotypes

**DOI:** 10.3390/cells11010129

**Published:** 2021-12-31

**Authors:** Concetta De Filippis, Barbara Napoli, Laura Rigon, Giulia Guarato, Reinhard Bauer, Rosella Tomanin, Genny Orso

**Affiliations:** 1 Laboratory of Diagnosis and Therapy of Lysosomal Disorders, Department of Women’s and Children’s Health, University of Padova, Via Giustiniani 3, 35128 Padova, Italy; concetta.defilippis@studenti.unipd.it; 2 Fondazione Istituto di Ricerca Pediatrica “Città della Speranza”, Corso Stati Uniti 4, 35127 Padova, Italy; laura.rigon@unipd.it; 3 Laboratory of Molecular Biology, Scientific Institute, IRCCS Eugenio Medea, Via Don Luigi Monza 20, Bosisio Parini, 23842 Lecco, Italy; barbaranapoli89@gmail.com; 4 Department of Pharmaceutical and Pharmacological Sciences, University of Padova, Via Marzolo 5, 35131 Padova, Italy; giulia.guarato@studenti.unipd.it; 5 Molecular Developmental Biology Unit, Life & Medical Sciences Institute (LIMES), University of Bonn, Carl-Troll-Straße 31, 53115 Bonn, Germany; unb30019@uni-bonn.de

**Keywords:** lysosomal storage disorders, mucopolysaccharidosis type I, MPS I, Hurler syndrome, *Drosophila melanogaster*, fly models, RNAi, IDUA, *D-idua*

## Abstract

Deficit of the IDUA (α-L-iduronidase) enzyme causes the lysosomal storage disorder mucopolysaccharidosis type I (MPS I), a rare pediatric neurometabolic disease, due to pathological variants in the *IDUA* gene and is characterized by the accumulation of the undegraded mucopolysaccharides heparan sulfate and dermatan sulfate into lysosomes, with secondary cellular consequences that are still mostly unclarified. Here, we report a new fruit fly RNAi-mediated knockdown model of a *IDUA* homolog (*D-idua*) displaying a phenotype mimicking some typical molecular features of Lysosomal Storage Disorders (LSD). In this study, we showed that *D-idua* is a vital gene in *Drosophila* and that ubiquitous reduction of its expression leads to lethality during the pupal stage, when the precise degradation/synthesis of macromolecules, together with a functional autophagic pathway, are indispensable for the correct development to the adult stage. Tissue-specific analysis of the *D-idua* model showed an increase in the number and size of lysosomes in the brain and muscle. Moreover, the incorrect acidification of lysosomes led to dysfunctional lysosome-autophagosome fusion and the consequent block of autophagy flux. A concomitant metabolic drift of glycolysis and lipogenesis pathways was observed. After starvation, *D-idua* larvae showed a quite complete rescue of both autophagy/lysosome phenotypes and metabolic alterations. Metabolism and autophagy are strictly interconnected vital processes that contribute to maintain homeostatic control of energy balance, and little is known about this regulation in LSDs. Our results provide new starting points for future investigations on the disease’s pathogenic mechanisms and possible pharmacological manipulations.

## 1. Introduction

Mucopolysaccharidosis type I (MPS I) (MIM #607015) is a lysosomal storage disorder (LSD) caused by mutations in the gene coding for the lysosomal enzyme α-L-iduronidase (IDUA), one of the lysosomal hydrolases responsible for the degradation of the glycosaminoglycans (GAG) heparan sulfate and dermatan sulfate. Thus, in case of IDUA deficit, these GAG progressively accumulate into lysosomes and lead to a chronic and progressive dysfunction of the cells, tissues, and organs of many systems, including the brain, in the severe forms [1,2]. Common features of the disease include skeletal deformities, recurrent ear and nose infections, cardiac valve disease, inguinal and umbilical hernias, facial dysmorphisms, hepatosplenomegaly, and, in the severe forms, developmental delay with progressive deterioration [3]. Clinically, it is characterized by a spectrum of phenotypes, while conventionally we distinguish three forms of the disease, Hurler, Hurler-Scheie, and Scheie syndromes, at decreasing severity. Biochemically, the three forms are indistinguishable, with all being characterized by the lack of IDUA enzyme activity and elevated GAG deposits [3].

Treatments consist of palliative and supportive care, hematopoietic stem cell transplantation (HSCT), and enzyme replacement therapy (ERT). Although effective in treating the neurological pathology, HSCT is most successful when carried out within the first 9 months of life and presents a high risk/benefit ratio [4]. Instead, ERT presents lower risks but fails to cross the blood-brain barrier and, therefore, to reach the central nervous system (CNS) [4,5,6,7].

Three murine models of MPS I were generated, starting in 1997 [8,9,10,11,12]. Overall, they mimic the characteristics of the human biochemical and clinical pathology [8,12,13,14,15,16,17], with altered facial features, skeletal abnormalities, locomotor defects, hepatosplenomegaly, progressive heart disease, deficits in long-term memory, and GAG accumulation in the urine and in several organs [8,12,13,14,15,16,17]. Microscopically, enlargement of lysosomes, neuroinflammation, synaptic dysregulation, and alterations of oxidative markers were observed [10,11,18]. Although the mouse remains the most used model for studies evaluating therapeutic efficacy, it is less commonly used for evaluations conducted to understand molecular pathogenetic aspects, mainly due to the experimental timing required, the elevated costs of colony maintenance, and the ethical issues involved, all factors limiting the sample size tested. Thus, up to the present time, such mechanisms underlying the pathogenesis remain poorly understood, especially those concerning the neurological aspects.

In the last few years, many neurodegenerative diseases, including some LSDs, have been modeled in *Drosophila* [19,20,21,22,23,24]. Our group has recently published a wide examination of these models [25]. The fruit fly models allowed, in most cases, to highlight the pathogenic mechanisms underlying the diseases, due to the simplicity in handling and the genetic manipulation tools available and provided good models for translational research. In particular, most of the fruit fly models generated to study lysosomal disorders, although not completely characterized in terms of molecular pathways and pathological mechanisms, share similar behavioral dysfunctions, such as a reduced lifespan, locomotor deficits, and neuronal cell death, highlighting the potential of these invertebrate models to study the neurological mechanism often associated with LSD disorders. On the contrary, as detailed below, other *Drosophila* models have been deeply analyzed and, at cellular level, alterations of vesicular trafficking, inhibition of autophagosome-lysosome fusion, and defects of autophagosome formation/accumulation seem to be common mechanisms. Indeed, lysosomal storage materials have a negative impact on the flux through autophagy and an increasing number of LSDs are described to have autophagy impairment [25], as was observed in the *Drosophila* models of mucolipidosis type IV, mucopolysaccharidosis type IIIA, and Gaucher disease. The *Drosophila* model of mucolipidosis type IV showed an increased number of lysosomes and an increased storage of lipofuscin. The latter is a clear sign of disrupted autophagy [23]. The *Drosophila* model of mucopolysaccharidosis type IIIA showed a disruption of vesicular trafficking, with impaired autophagic activity [24]. Inhibition of autophagy leads to an accumulation of damaged mitochondria and to oxidative stress that can induce apoptosis and, therefore, neurodegeneration, as observed in mucolipidosis type IV and mucopolysaccharidosis type VII [19,23]. Other pathways are thought to be implicated in LSDs. For example, some *Drosophila* models of LSDs were associated with mitochondrial dysfunction, oxidative stress, and UPR activation [23,26,27]. Accumulation of acidic vesicles containing the undegraded sphingolipids was observed in the model, suggesting lysosomal dysfunction, a block in the autophagic pathway, and the accumulation of defective mitochondria [28]. Moreover, defects of the degradative system are also linked to metabolic changes in the same *Drosophila* model of mucolipidosis type IV described above [23], as an abnormal transport of the lipids to the lysosomes, loss of activity of AMPK (5′ AMP-activated protein kinase), and the inactivation of TRPLM1 through phosphorylation were observed [29]. A better understanding of the altered metabolic pathways would allow for a more accurate overview of the pathological mechanisms, the possible targets of pharmacological modulation, and ad hoc nutritional interventions.

Our MPS I *Drosophila* model was generated by using the RNA interference (RNAi) approach. We centered our analysis on flies with a selective downregulation of *D-idua* in neurons and glial cells, observing a progressive locomotor impairment. Moreover, as *D. melanogaster* is a model system used to study autophagy [30], as previously seen in other MPSs and LSDs [31,32,33,34] (reviewed in [35]), we analyzed this pathway to better characterize it in this new model of MPS I. Finally, we analyzed the metabolic changes associated with the enzyme reduction.

This novel fly model may help to better understand the pathogenic mechanisms underlying the disease, to find new markers of the pathology, to identify new therapeutic targets, and to evaluate novel therapies.

## 2. Materials and Methods

### 2.1. Drosophila Strains

All *Drosophila* Strains are reported in Table 1. Fly stocks were raised on standard medium [36] and in standard light and temperature conditions. Gal4/UAS crossings were performed at 28 °C. The recombinant line UAS-GFP-Lamp1;mcherry-Atg8a was generated in our laboratory. The control genotype (w^1118^) was crossed with the drivers. For each experiment, groups comprising 50% of males and 50% of females were analyzed.

### 2.2. RNA Extraction and RT-PCR

The relative *IDUA* expression levels were determined by quantitative real-time PCR (qRT-PCR). For each evaluation, total RNA was isolated from five third instar larvae, using the GRS FullSample Purification Kit (GriSP, Lda, Porto, Portugal), according to the manufacturer’s instructions. Real-time PCR was performed with the Eco Real-Time PCR System (Illumina Inc., San Diego, CA, USA), using the One-Step SYBR^®^ Prime Script TM RT-PCR Kit II (Takara-Clontech, Kusatsu, Japan) as in [37]. The housekeeping *Rp49* gene was used to normalize the data. Each biological sample was loaded in triplicate and the data provided represent the mean values of the three independent biological replicates. The *Drosophila* primer pairs used are reported in Table 2.

### 2.3. IDUA Activity Assay

Twenty third instar larvae were homogenized in 0.9% NaCl plus protease inhibitors (Roche, 05892791001). After centrifugation, supernatants were recovered, and the protein concentration was determined (Bradford Reagent, # 39222.03, Serva, Heidelberg, Germany). IDUA activity was evaluated by a fluorometric assay using the human substrate 4-methylumbelliferyl α-L-iduronide (4MU-iduronide) (Glycosynth #44076, Warrington, UK), as previously described [38]. The final IDUA activity is given as nanomoles of substrate hydrolyzed in 1 h per mg of protein.

### 2.4. GAG Analysis

Third instar larvae were lyophilized, homogenized in 0.9% NaCl + 0.2% Triton X-100 (PanReac AppliChem GmbH, Darmstadt, Germany) using a Polytron^®^PT1200E Disperser (Kinematica AG, Luzern, Switzerland), stirred overnight at 4 °C, centrifuged at 1000× *g* for 5 min, and the supernatant was recovered. Protein concentration was determined, and GAG content was measured using Björnsson’s protocol [39] with modifications, as previously described [40].

### 2.5. Eclosion Rate

Female virgins and males were placed in a vial in a 10:5 ratio and allowed to lay eggs for 48 h. Then, adults were discarded, and larvae were allowed to develop for 13 days. Pupae were counted and flies that successfully eclosed were scored. The data are expressed as the percent of eclosion (number of adult flies eclosed/number of pupae).

### 2.6. Immunohistochemistry

Third instar larvae, which were raised at 28 °C, were harvested and dissected, as previously described [41]. Afterwards, they were incubated with the primary antibodies rabbit anti-Ref(2)p (1:200, ab178440, Abcam plc, Cambridge, UK), mouse anti-Elav (1:100, 9F8A9, DSHB, Iowa City, IA, USA), and mouse anti-Repo (1:100, 8D12, DSHB, Iowa City, IA, USA) over-night at +4 °C. After washing, the larvae were incubated in the secondary antibody anti-Rabbit CyTM5 (1:500, Cat# 111-175-144) or anti-Mouse CyTM3 (1:500, Cat# 115-165-003) (Jackson ImmunoResearch Europe Ltd., Cambridge, UK) for 1 h at RT then washed and mounted on glass slides using Mowiol^®^ 4-88 (Cat# 324590, Sigma-Aldrich, St. Louis, MO, USA).

### 2.7. Lysotracker Assay/Staining

Third instar larvae were dissected in HL3, incubated for 10 min in LysoTracker^TM^ Red DND-99 (1:2000, L7528, Life Technologies, Thermo Fisher Scientific, Waltham, MA, USA) with a 20 µM glutamate solution, covered with a glass slide, and immediately photographed, as previously described [42].

### 2.8. Microscopy Imaging

Live images were acquired using a confocal microscope (Nikon D-ECLIPSE C1) equipped with a Nikon 60x/1.40 oil Plan-Apochromat objective using the Nikon EZ-C1 acquisition software. Fixed larvae were acquired using a ZEISS LSM 800 Confocal Laser Scanning Microscope (Carl Zeiss, Jena, Germany) equipped with a Zeiss 63x/1.4– Plan-Apochromat oil objective using the ZEN Blue acquisition software. Muscles and ventral nerve cords of ten third instar larvae per group were quantified and analyzed with ImageJ Fiji 1.52 software (NIH, Bethesda, MD, USA) [43].

### 2.9. Microscopy Analysis

ImageJ was used to produce a maximum intensity projection of the stack. The UAS-GFP-Lamp1 strain was used to quantify the number and size of lysosomes, whereas the UAS-mcherry-Atg8a strain was used to quantify the number of autophagosomes. The thresholds of the GFP, mcherry/Lysotracker, and Cy5 signals were produced with the automatic threshold function. This allowed the elimination of the background signal, and the number of particles, as well as their area, were calculated using the ‘particle analysis’ tool. For the quantification of co-localized particles, ImageJ was used to threshold the red and green channels and count the GFP and mcherry/Lysotracker dots. The co-localization of the channels was calculated using the co-localization function of ImageJ. For each sample, two muscles and three Regions of Interest (ROI) of 350 μm^2^ per muscle were analyzed. For each brain, three ROI of 150 μm^2^ were analyzed. The percentage of acidified lysosomes was calculated as follows:

$$\frac{\mathrm{yellow}\;\mathrm{puncta}\;\left(\mathrm{Co}-\mathrm{loc}\;\mathrm{GFP}-\mathrm{Lamp}1-\mathrm{Lysotracker}\right)}{\mathrm{green}\;\mathrm{puncta}\;\left(\mathrm{GFP}-\mathrm{Lamp}1\right)}\times 100$$

The percentage of autolysosomes was calculated as follows:

$$\frac{\mathrm{yellow}\;\mathrm{puncta}\;\left(\mathrm{autolysosomes}\;\mathrm{co}-\mathrm{loc}\;\mathrm{GFP}-\mathrm{Lamp}1-\mathrm{mcherry}-\mathrm{Atg}8a\right)}{\mathrm{red}\;\mathrm{puncta}\;\left(\mathrm{autophagosomes}\;\mathrm{mcherry}-\mathrm{Atg}8a\right)}\times 100$$

The percentage of mature autophagosomes was calculated as follows:

$$\frac{\mathrm{red}\;\mathrm{puncta}\;\left(\mathrm{autophagosomes}+\mathrm{mature}\;\mathrm{autophagosomes}\;\mathrm{mcherry}-\mathrm{Atg}8a\right)-\mathrm{green}\;\mathrm{puncta}\;\left(\mathrm{autophagosomes}\;\mathrm{GFP}-\mathrm{Atg}8a\right)}{\mathrm{red}\;\mathrm{puncta}\;\left(\mathrm{mcherry}-\mathrm{Atg}8a\right)}\times 100$$

### 2.10. Climbing Assay

20 to 30 flies for each genotype were collected after eclosion and transferred to tubes containing fresh standard food, then tested twice a week. *Drosophilae* were placed in an empty tube and gently tapped to the bottom. The number of flies that reached or passed the line 2 cm from the bottom in 20 s was recorded as the percentage of flies able to climb the vial. Three separate and consecutive trials were performed, and the results were averaged. The experiment was repeated 10 times for each genotype [36].

### 2.11. Lifespan Assay

We collected newly eclosed animals and bred them at low density (<20 animals per vial) in standard conditions. Aging animals were transferred to new vials three times per week, with deaths scored. Lifespan plots were generated by calculating the percentage of survivorship and plotting viability as a function of time [36].

### 2.12. Starvation Assay

Ten third instar larvae per group were individually selected and placed in Petri plates containing a solution of 20% sucrose in PBS for 4 h.

### 2.13. Western Blot

For protein analysis, third instar larvae were homogenized and purified using the GRS FullSample Purification Kit (GriSP, Lda, Porto, Portugal), according to the manufacturer’s instructions. Proteins were quantified using the Bradford method (Serva, Heidelberg, Germany). For western blot analysis, 20 µg of extracted proteins were mixed with the LDS sample buffer 4x (Invitrogen, Waltham, MA, USA) and the reducing agent 10x (Invitrogen, Waltham, MA, USA), then boiled for 10 min at 75 °C. Next, they were loaded and electrophoresed in Bolt 4–12% gradient gel (Invitrogen, Waltham, MA, USA) and transferred onto PVDF membranes. The membranes were blocked in blocking buffer (Thermo Fisher Scientific, Waltham, MA, USA). Immunodetections were performed using the following antibodies: rabbit anti-Ref(2)p (1:1000, ab178440, Abcam plc, Cambridge, UK), rabbit anti-LC3 (1:2000, ABC974, Sigma-Aldrich, MO, USA), and mouse anti-β actin (1:5000, A5441, Sigma-Aldrich, MO, USA) as loading control. The secondary antibody anti-rabbit HRP was used at a concentration of 1:5000 (A16110, Invitrogen, MA, USA) and anti-mouse HRP (A4416, Sigma-Aldrich, MO, USA) was used at a concentration of 1:20,000. The signals were detected using the Western blotting luminol reagent by Santa Cruz Biotechnology, Inc. (Dallas, TX, USA). The protein bands were detected using the iBright FL1500 Imaging System (Thermo Fisher Scientific, Waltham, MA, USA) and densitometry measurements of the western blot images were performed using Fiji software. Three independent biological replicates were analyzed in double.

### 2.14. Statistical Analysis

The statistical analysis was conducted using GraphPad Prism Software. Student’s *t*-tests, one-way ANOVAs or two-way ANOVAs with Tukey’s *post hoc* tests were applied. Error bars represent standard errors of the means. *p* < 0.05 was taken as the threshold for the statistical significance.

## 3. Results

### 3.1. Identification of CG6201 as an Active Orthologue of IDUA in Drosophila

**Figure 1:**
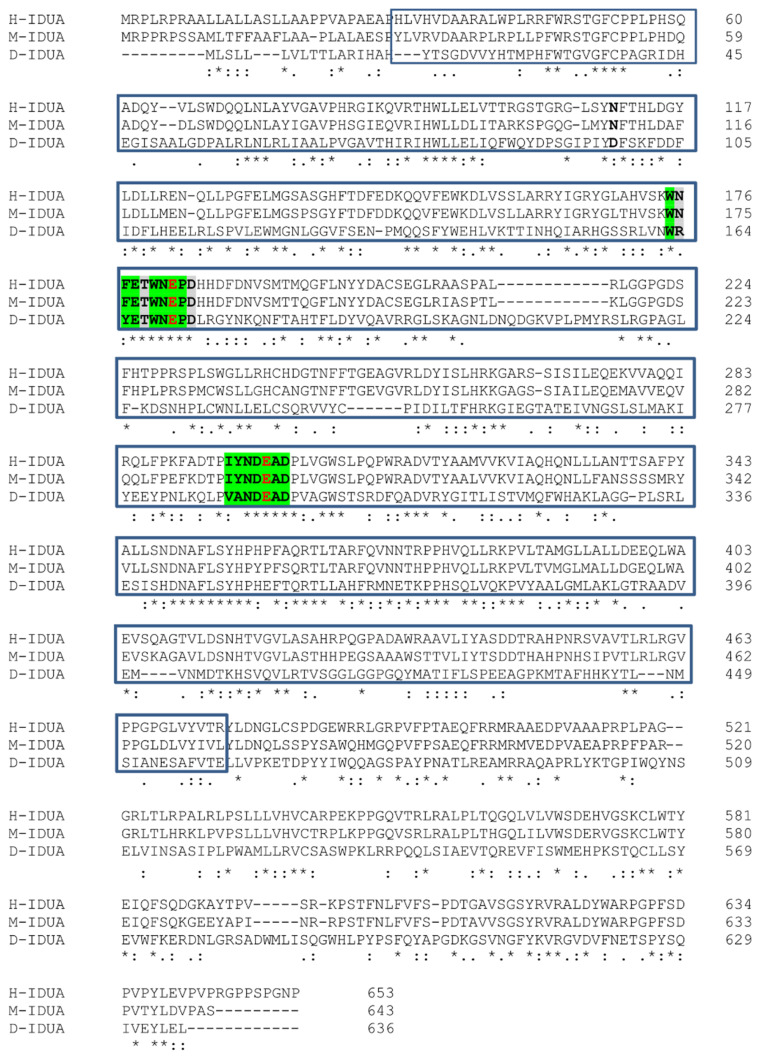
Human, mouse, and *Drosophila* IDUA protein alignment. Sequence alignments of human, mouse, and *Drosophila* IDUA proteins. The glycosyl hydrolase domain is boxed in blue, highlighted in green are the regions flanking the predicted nucleophiles and acid/base catalysts, and the acid/base residue and nucleophile are marked in red. * (asterisk) indicates positions which have a single, fully conserved residue. (colon) indicates conservation between groups of strongly similar properties. (period) indicates conservation between groups of weakly similar properties.

As reported in the *Drosophila* Flybase (Flybase. Available online: https://www.flybase.org/ (accessed on 22 December 2021)) the homologue of the human *IDUA* (accession number P35475) is the annotated gene *CG6201* (accession number Q9VKJ8), which exhibits 30% identity and 47% similarity at the aminoacidic level with the human and mouse (accession number P48441) homologs (Figure 1). The aminoacidic residues of the two active sites were conserved between the species (Figure 1).

As the CG6201 coding protein was structurally conserved, we tested whether CG6201 has the functional capability to catalyze the human substrate 4MU-iduronide. For this purpose, wild-type larvae at third instar stage and adult flies were evaluated for enzymatic activity using the human substrate and showed 4.69 and 3.57 nmol/1 h/mg protein activity, respectively. These data showed that in addition to the structural similarity, CG6201 maintains the functional feature of IDUA enzymatic activity, and, thus, we concluded that *CG6201* encodes for a protein which is an active orthologue of IDUA in *Drosophila*. We, therefore, proposed to name *CG6201* as *D-idua* for this work.

### 3.2. D-idua Is an Essential Gene in Drosophila

**Figure 2:**
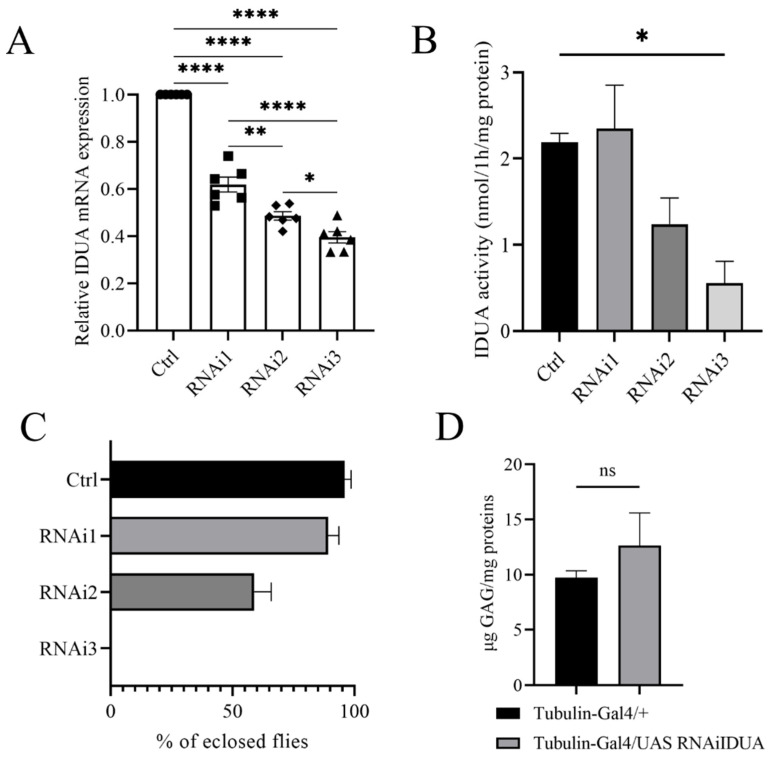
Characterization of the D-idua RNAi lines. (**A**) qRT-PCR on third instar larvae down-regulated for the *D-idua* gene with the driver Tubulin-Gal4 (one-way ANOVA with Tukey’s *post hoc* test. * *p* < 0.05; ** *p* < 0.01; **** *p* < 0.0001). Data are the results of three different larvae extracts, each of them analyzed twice, in triplicate. (**B**) D-idua activity in III instar larvae when *D-idua* was ubiquitously down-regulated with the driver Tubulin-Gal4. Data are the result of three different larvae extracts, each of them analyzed twice, in duplicate. (two-tailed Student’s *t*-test * *p* < 0.05) (**C**) % of eclosed flies when *D-idua* was down-regulated with the driver Tubulin-Gal4 in the three different UAS IDUA strains. (**D**) GAG analysis in III instar larvae when *D-idua* was ubiquitously down-regulated with the driver Tubulin-Gal4. Data are the result of three different larvae extracts, each of them analyzed twice, in duplicate (two-tailed Student’s *t*-test). All data are expressed as means ± SEM. Asterisks indicate a statistically significant difference.

To evaluate the biological function of D-idua we analyzed the in vivo effects associated with *D-idua* downregulation. The genetic reduction was assessed by knocking-down *D-idua* using the RNAi approach and the UAS-Gal4 system [44]. The inspection of the VDRC (Vienna Drosophila Stock Center) highlighted three UAS RNAi lines available: 13244/GD hereafter referred to as D-idua^RNAi1^, 103771/KK (D-idua^RNAi2^), and 13245/GD (D-idua^RNAi3^) (Table 1). Using Tubulin-Gal4, a strong ubiquitous driver line, in combination with the three RNAi lines, *D-idua* knockdown was shown to be effective to a different extent. In particular, with D-idua^RNAi1^, D-idua^RNAi2^, and D-idua^RNAi3^ in combination with Tubulin-Gal4, expression was reduced by 39%, 53%, and 61%, respectively (Figure 2A). The enzymatic activity was correlated with *D-idua* mRNA expression, with no changes in the IDUA activity when D-idua^RNAi1^ was used, a reduction to half with D-idua^RNAi2^, and a reduction to one-third with D-idua^RNAi3^ (Figure 2B). Consequently, we observed different lethality levels: D-idua^RNAi1^ expression showed no obvious altered phenotype, with an eclosion rate similar to that of control flies; D-idua^RNAi2^ expression caused partial lethality at the pupal stage, with almost 70% of escapers, and D-idua^RNAi3^ expression caused complete lethality at the pupal stage, with no flies reaching the adult stage (Figure 2C). Since *D-idua* knockdown was most effective and showed the strongest in vivo phenotype with D-idua^RNAi3^, this strain was selected for all further analyses. We next analyzed GAG deposits, a hallmark of MPS, commonly registered in human cells, in the MPS I mouse model [38], as well as in the *Drosophila* models of other MPS [19,24]. Interestingly, we observed an increased, although not significant, GAG level in comparison to controls (Figure 2D). This might be due to the residual enzyme activity still present in the knockdown model or due to the time of analysis in the larval stage, where GAG deposits may not be significantly established yet (Figure 2D).

These results showed that D-idua is essential in *D. melanogaster* during development and that a certain threshold level of D-idua is required to avoid defects in the correct development.

### 3.3. Effects of Ubiquitous and Tissue-Specific D-idua Downregulation on Lethality, Lifespan, and Locomotion

**Figure 3:**
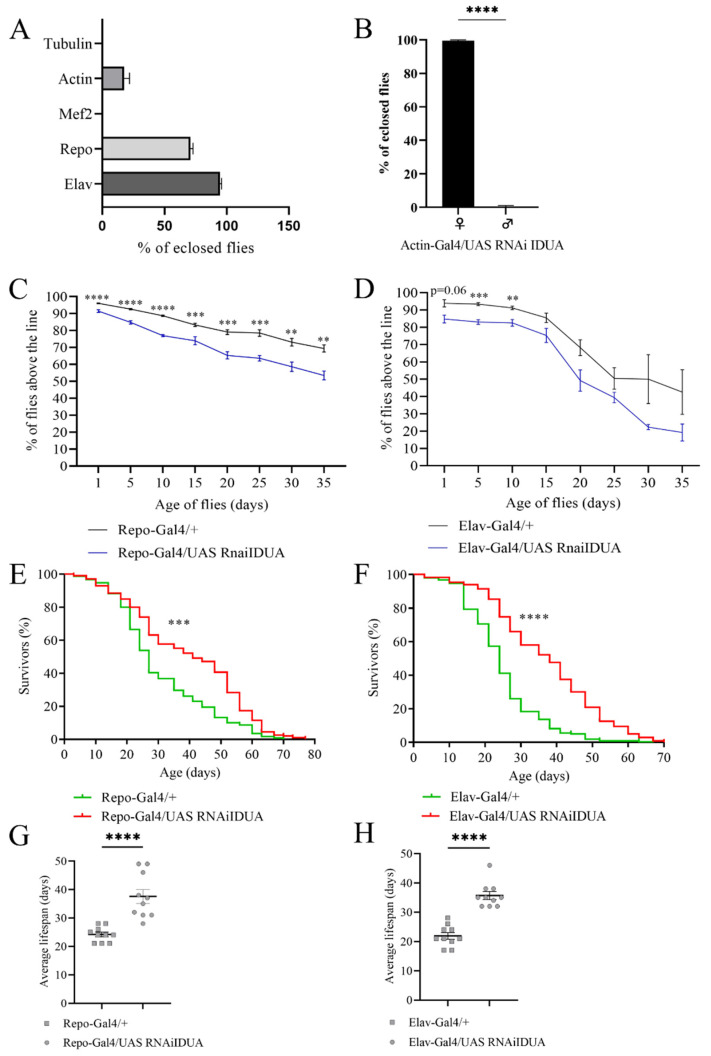
Effects of tissue-specific downregulation of *D-idua*. (**A**) % of eclosed flies when *D-idua* was down-regulated with different ubiquitous and tissue-specific drivers. (**B**) % of female and male flies eclosed when *D-idua* was down-regulated with the ubiquitous driver Actin-Gal4 (two-tailed Student’s *t*-test **** *p* < 0.0001). Climbing activity of adult flies when *D-idua* was down-regulated in (**C**) glial cells and (**D**) neurons (multiple unpaired *t*-tests ** *p* < 0.01; *** *p* < 0.001; **** *p*<0.0001). *n* = 250–300 flies/group. The lifespan of adult flies when *D-idua* was down-regulated in (**E**) glial cells and (**F**) neurons (Long-rank (Mantel–Cox) test *** *p*<0.001; **** *p* < 0.0001). *n* = 200–250 flies/group. The average lifespan of adult flies when *D-idua* was down-regulated in (**G**) glial cells and (**H**) neurons (two-tailed Student’s *t*-test **** *p* < 0.0001).

To characterize the impact of reduced D-idua activity in *Drosophila,* we a performed tissue-specific downregulation of the gene. When *D-idua* was downregulated in neurons (Elav-Gal4), we observed no lethality (96.8% eclosion rate), whereas downregulation in glial cells (Repo-Gal4) led to partial lethality at the pupal stage, with about 73% of flies reaching the adult stage. After downregulation of *D-Idua* expression using the ubiquitous driver Actin-Gal4, a weaker driver compared to Tubulin-Gal4 [45], we observed about 20% of flies reaching adult stage (Figure 3A). Of note, all the adult flies that eclosed were females, which may be due to the higher *D-idua* expression in females compared to males (data from RNAseq on Flybase. Available online: https://www.flybase.org/. (accessed on 22 December 2021)) (Figure 3B).

Lastly, downregulation of *D-idua* in muscles with the Mef2-Gal4 driver line caused complete lethality at the pupal stage, suggesting that *D-idua* is necessary for the correct development of muscle tissue.

To assess whether the enzyme deficit in neurons and glial cells causes locomotor impairment, we tested Elav-Gal4/UAS D-idua^RNAi3^ and Repo-Gal4/UAS D-idua^RNAi3^ adult flies using the negative geotaxis assay [46]. As shown in Figure 3C,D, both tissue-specific downregulations caused a constant locomotor deficit compared to control flies, starting from day 1 after eclosion when *D-idua* was downregulated in glial cells (Repo-Gal4) (Figure 3C) and from day 5 when *D-idua* was downregulated in neurons (Elav-Gal4) (Figure 3D). The locomotion deficit was more pronounced in flies with reduced expression of *D-idua* in glial cells. In contrast, the reduction of *D-idua* in neurons lead to a significant difference only during the first 10 days, whereas no significant differences were observed later on between control and *D-idua* flies. Besides causing a locomotor deficit, this progressive decline may also be indicative of a neurological impairment. Despite the induced locomotor impairment, these flies unexpectedly displayed a longer lifespan compared to controls. In the viability assay, we observed that *D-idua* downregulation in glial cells increased adult lifespan, where 50% of flies were still alive after 40 days, compared to 25 days for controls (Figure 3E–G). The same results were obtained with the downregulation of *D-idua* in neurons, where the median lifespan of adult flies was 35 days after eclosion (Figure 3F–H).

### 3.4. D-idua Downregulation Leads to Lysosomal Defects

**Figure 4:**
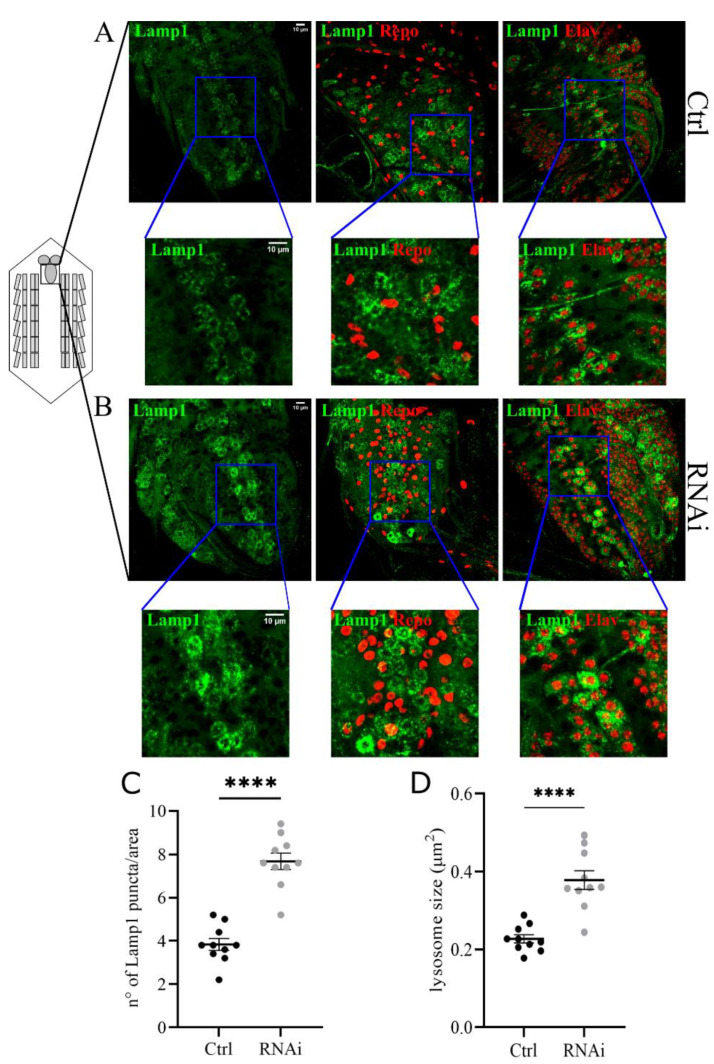
Downregulation of *D-idua* leads to the accumulation of enlarged lysosomes in the central nerve cord. Representative confocal images of central nerve cords expressing the marker GFP-Lamp1 in (**A**) control and (**B**) RNAi III instar larvae and the relative quantification of the (**C**) number and (**D**) size of lysosomes. *n* = 10 larvae/group. All data are expressed as means ± SEM. Asterisks indicate a statistically significant difference with respect to control (two-tailed Student’s *t*-test **** *p* < 0.0001). Genotypes of samples: Ctrl = Tubulin-Gal4; UAS GFP-Lamp1/+; RNAi = Tubulin-Gal4; UAS GFP-Lamp1/UAS D-idua^RNAi3^. The size and number of lysosomes were automatically calculated with the software ImageJ in a ROI (area) of 150 µm^2^, as specified in the materials and methods section.

**Figure 5:**
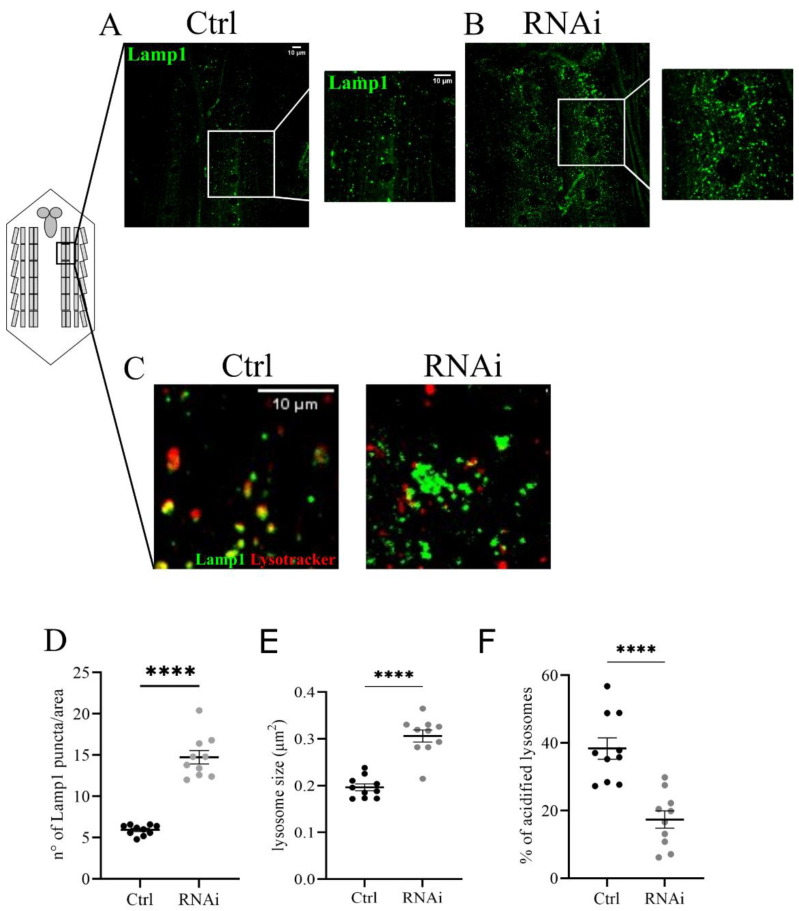
Lysosomal defects associated with a reduction of *D-idua* in muscles. Representative confocal images of muscles expressing the marker GFP-Lamp1 in (**A**) control and (**B**) RNAi III instar larvae and the relative quantification of the (**D**) number and (**E**) size of lysosomes. (**C**) Representative confocal images of III instar larvae muscles expressing the marker GFP-Lamp1 and stained with the probe Lysotracker red. (**F**) Quantification of correctly acidified lysosomes in the muscles of III instar larvae. *n* = 10 larvae/group. All data are expressed as means ± SEM. Asterisks indicate a statistically significant difference from the control (two-tailed Student’s *t*-test **** *p* < 0.0001). Genotypes of samples: Ctrl = Tubulin-Gal4; UAS GFP-Lamp1/+; RNAi = Tubulin-Gal4; UAS GFP-Lamp1/UAS D-idua^RNAi3^. The number and size of lysosomes were automatically calculated with the software ImageJ in a ROI (area) of 350 µm^2^, as specified in the materials and methods section.

We focused on lysosome morphology and the functional acidification of these organelles. To analyze changes in the size and number of lysosomes we co-expressed the UAS-GFP-Lamp1 (lysosomal acidic membrane protein isoform 1 fused to the green fluorescent protein) marker and the D-idua^RNAi3^ line using the Tubulin-Gal4 driver (Figure 4A,B). The analysis of the ventral nerve cord of third instar larvae showed a significant increase in the number and size of lysosomes compared to controls (Figure 4C,D). The same phenotype was also observed in muscles with reduced levels of *D-idua*, with a similar increase in the number and size of lysosomes (Figure 5D,E). Since lysotracker can easily cross the membrane in muscles, we performed live image tracing of the lysotracker signal to quantify acidic organelles. Quantification of the muscle tissue expressing the lysosomal marker GFP-Lamp1 and the lysotracker probe demonstrated that less than 20% of lysosomes were correctly acidified in D-idua^RNAi3^ muscle flies compared to 40% of controls (Figure 5F). Alterations in lysosome homeostasis, accompanied with a decrease of their acidification, was previously shown in the MPS I mouse model [47].

### 3.5. The Inhibition of Autophagy Flux by D-idua Reduction Is Nutrient-Sensitive and Ameliorates in Starvation

The abnormal lysosomal function, as well as an inefficient degradative capability of the lysosomes, have a negative impact on the flux through the autophagy pathway [48].

**Figure 6:**
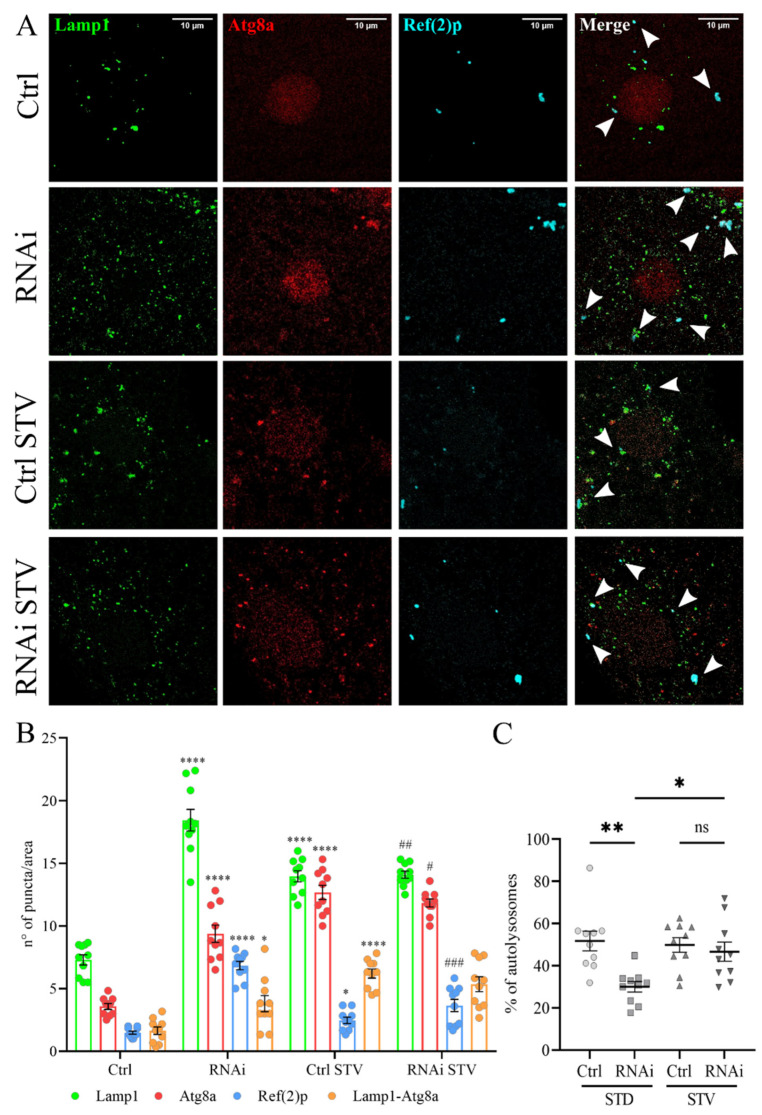
Reduction of *D-idua* induces defective autophagosome fusion. (**A**) Representative confocal images of III instar larvae muscles expressing the markers GFP-Lamp1, mcherry-Atg8a, and Ref(2)p. (**B**) Number of lysosomes, autophagosomes, and Ref(2)p puncta in III instar larvae muscles. Asterisks indicate a statistically significant difference vs. Ctrl and hash marks indicate a statistically significant difference vs. RNAi (two-way ANOVA with Tukey’s *post hoc* test. * *p* < 0.05; **** *p* < 0.0001; # *p* < 0.05; ## *p* < 0.01; ### *p* < 0.001). (**C**) % of autolysosomes in III instar larvae muscles. Asterisks indicate a statistically significant difference (one-way ANOVA with Tukey’s *post hoc* test. * *p* < 0.05; ** *p* < 0.01); *n* = 10 larvae/group. All data are expressed as means ± SEM. Genotypes of samples: Ctrl = Tubulin-Gal4; UAS GFP-Lamp1, mcherry-Atg8a/+; RNAi = Tubulin-Gal4/UAS GFP-Lamp1, mcherry-Atg8a/UAS D-idua^RNAi3^. STD indicates standard conditions; STV indicates a starvation of 4 h. Arrows indicate autophagosomes.

To examine the role of *D-idua* in autophagy we investigated the capability of autophagosomes to fuse to lysosomes, as well as the autophagy flux, in control and in D-idua^RNAi3^ larval muscles, comparing standard and starvation conditions. Starvation is a well-known cellular stimulus for autophagy activation, leading to a marked increase in the number and size of lysosomes. Autophagosome-lysosome fusion was measured by the simultaneous expression of mCherry-Atg8a, known to mark all autophagy structures (phagophores, autophagosomes, and autolysosomes) and the endo/lysosomal markers GFP-Lamp1 using Tubulin-Gal4 (Figure 6A). The ratio of autolysosome number (mCherry-Atg8a positive and GFP-Lamp1 positive) and autophagosome vesicles (mCherry-Atg8a positive and GFP-Lamp1 negative) is a measure of the dynamic scenario of autophagosome-lysosome fusion events [49,50]. Furthermore, we stained the samples with the Ref(2)p (*Drosophila* homologue of P62) antibody as an additional marker to evaluate the cellular degradative system [51]. As already reported in other studies [50], we found that the total number of autophagic vesicles (red, mCherry-Atg8a) increased after starvation in control larvae compared to fed ones, while the ratio between autolysosome and autophagosomes remained stable (Figure 6B), indicating a successful delivery of autophagosomes to the lysosomes by mCherry-Atg8a-labeled autolysosomes. When the same analysis was performed in D-idua^RNAi3^ larvae, we detected, already under fed conditions, a significant increase in mcherry-Atg8 puncta that could be due to the activation of autophagy and/or to the block of autophagy flux. However, the percentage of autophagosomes that fused to lysosomes was drastically diminished by the reduction of *D-idua*, suggesting that a threshold value of *D-idua* is required for autophagosome-lysosome fusion (Figure 6C). Immunostainings also revealed Ref(2)p/p62-positive aggregates in the cytoplasm of D-idua^RNAi3^ compared to controls, in line with a defective autophagic cargo clearance.

In contrast, we observed a significant increase in the fusion of lysosomes to autophagosomes in starved D-idua^RNAi3^ larvae (54% of autolysosomes in starvation vs. 26% in fed larvae), making them comparable to control larvae, where about 55% of autophagosomes were fused with lysosomes in both feeding and starvation conditions (Figure 6C). Moreover, short-term starvation reduced the number of lysosomes and of Ref(2)p-positive autophagosomes, the latter being suggestive of a reduction of the autophagic block.

**Figure 7:**
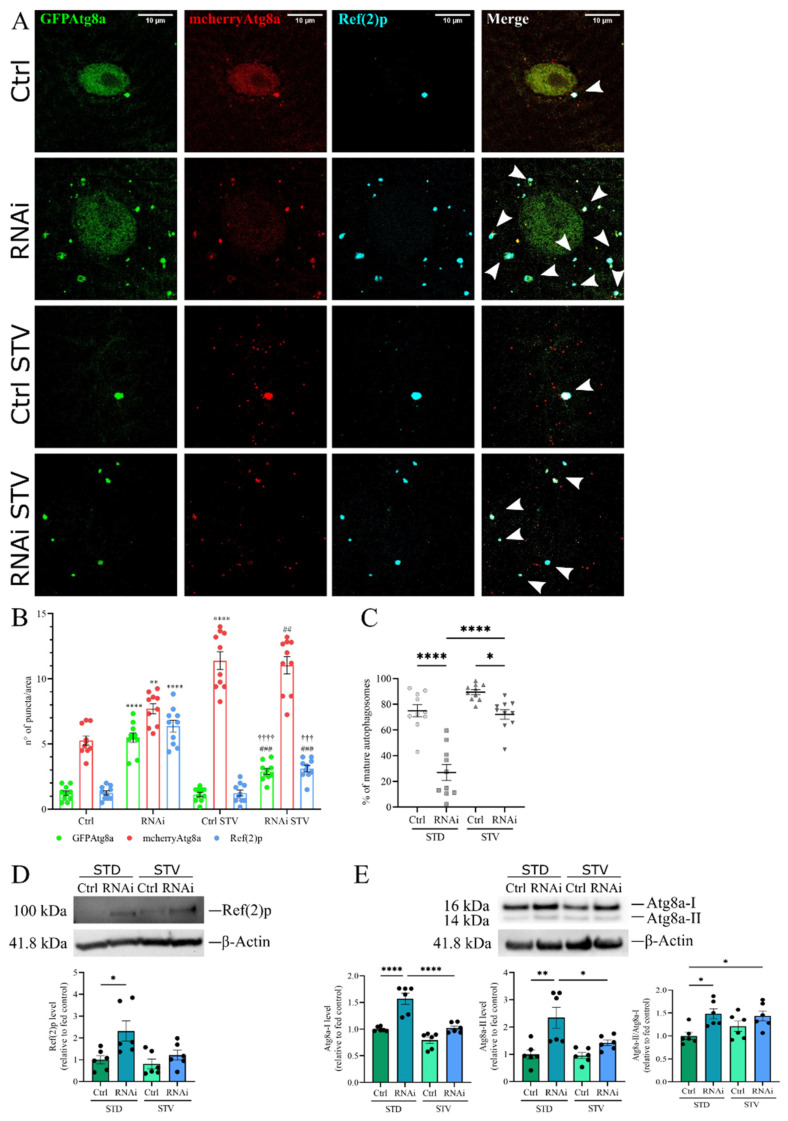
Reduction of *D-idua* leads to the accumulation of defective autophagic structures. (**A**) Representative confocal images of III instar larvae muscles expressing the markers GFP-mcherry-Atg8a and Ref(2)p. (**B**) Number of autophagosomes, mature autophagosomes, and Ref(2)p puncta in III instar larvae muscles. Asterisks indicate a statistically significant difference vs. Ctrl, hash marks indicate a statistically significant difference vs. RNAi, and crosses indicate a statistically significant difference from Ctrl STV (two-way ANOVA with Tukey’s *post hoc* test. ** *p* < 0.01; **** *p* < 0.0001; ## *p* < 0.01; ### *p* < 0.001; ††† *p* < 0.001; †††† *p* < 0.0001). (**C**) % of mature autophagosomes in III instar larvae muscles. Asterisks indicate a statistically significant difference (one-way ANOVA with Tukey’s *post hoc* test. * *p* < 0.05; **** *p* < 0.0001); *n* = 10 larvae/group. Genotypes of samples: Ctrl = Tubulin-Gal4; UAS GFP-mcherry-Atg8a/+; RNAi = Tubulin-Gal4/UAS GFP-mcherry-Atg8a/UAS D-idua^RNAi3^. (**D**) Western blot of Ref(2)p and the relative quantification (one-way ANOVA with Tukey’s *post hoc* test. * *p* < 0.05). (**E**) Western blot of Atg8a and the relative quantification (one-way ANOVA with Tukey’s *post hoc* test. * *p* < 0.05; ** *p* < 0.01; **** *p* < 0.0001). Genotypes of samples: Ctrl = Tubulin-Gal4;/+; RNAi = Tubulin-Gal4/UAS D-idua^RNAi3^. All data are expressed as means ± SEM. STD indicates standard conditions; STV indicates a starvation of 4 h. Arrows indicate autophagosomes.

We repeated the experiment in muscles using the tandem mCherry-GFP-Atg8a reporter, to measure the autophagy flux by taking advantage of the differential pH sensitivity of GFP and mCherry (Figure 7A). The mCherry signal is maintained when autophagosomes fuse with lysosomes, whereas the GFP is quenched by the acidic environment; thus, the analysis discriminates between red fused autophagosomes (mCherry positive-GFP negative) and the yellow non-fused autophagosomes or non-acidified vesicles (mCherry positive-GFP positive). The ratio between red and yellow was used to estimate the transition from autophagosome to autolysosome.

In control larvae grown in standard food, autophagosomes were barely detected, whereas autophagy induction by starvation led to the formation of acidic autolysosomes, which could be visualized only in the red channel, due to GFP quenching. In fed D-idua^RNAi3^ larvae, we observed the formation of non-acidified (red and green fluorescent) autophagic structures, suggesting that autophagosome clearance was severely blocked (Figure 7B). As seen in the lysosome-autophagosome fusion assay (Figure 6), after starvation the autophagosome level in *D-idua*-downregulated larvae was almost similar to controls, with about 70% of mature autophagosomes. In contrast only about 50% of autophagosomes were detected in standard food conditions (Figure 7C). Moreover, Ref(2)p aggregates diminished, mimicking control larvae samples. Quantification of Ref(2)p protein levels confirmed the immunostaining results. As shown in Figure 7D, there was an accumulation of Ref(2)p in *D-idua* third instar larvae as compared to controls. Upon starvation, Ref(2)p returned to control levels, whereas Ref(2)p levels in control larvae remained unchanged under both nutrient conditions.

During the elongation phase of autophagy, the cytosolic form of Atg8a, Atg8a-I, is conjugated to phosphatidylethanolamine to form Atg8a-II, which is specifically targeted to the autophagosome membrane, where it remains until fusion with lysosomes, where it is delipidated and recycled. The turnover of Atg8a is, therefore, considered a good marker for studying autophagy [52]. Western blot analysis showed that Atg8a-I and Atg8a-II accumulated in *D-idua* third instar larvae as compared to controls. Increased levels of Atg8a-II suggest an increased number of autophagosomes; however, the simultaneous increase of Atg8a-I could also indicate a block in autophagic flux [53]. In addition, analysis of the Atg8a-II/Atg8a-I ratio confirmed increased autophagosome formation and an autophagy block in *D-idua* third instar larvae (Figure 7E). All these markers are restored to normal levels under starvation conditions, further confirming our immunofluorescence assay data.

Our data emphasize that *D-idua* is a positive regulator of autophagy flux and a modulator of endolysosomal degradative system.

### 3.6. D-idua Reduction Is Accompanied by Switches in Metabolic Gene Expression

**Figure 8:**
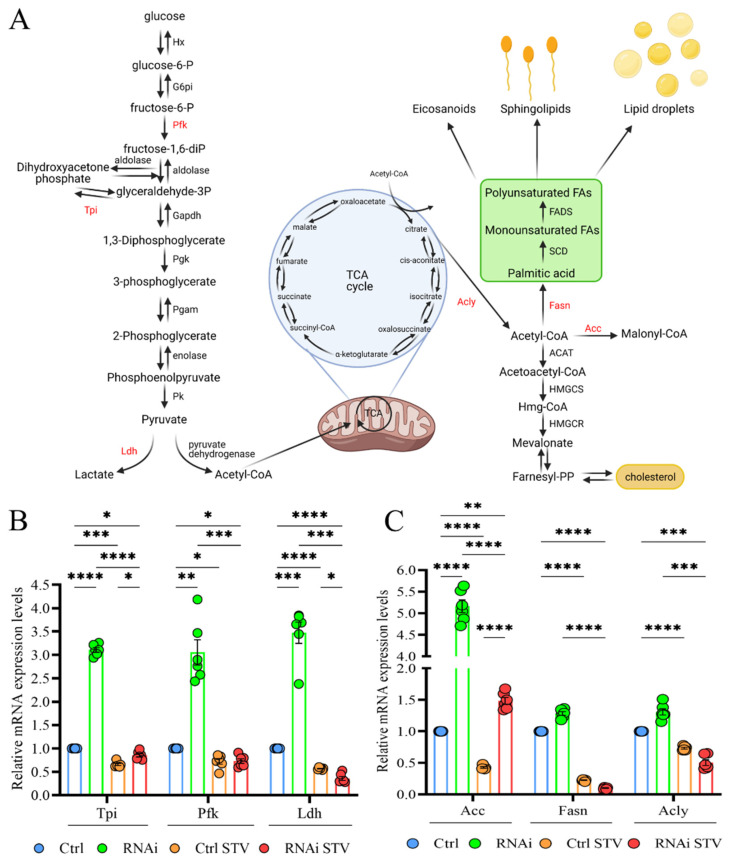
Downregulation of *D-idua* results in dysregulated glycolysis and lipogenesis. (**A**) Schematic representation of the crosstalk between glycolysis (on the left) and lipogenesis (on the right). The genes considered in our analyses are marked in red. (Created with BioRender. Available at: http://www.biorender.com/. accessed on 23 November 2021). (**B**) Relative mRNA expression levels of genes involved in glycolysis. (**C**) Relative mRNA expression levels of genes involved in lipogenesis. Data are the results of three different larvae extracts, each of them analyzed twice, in triplicate. All data are presented as means ± SEM. Asterisks indicate a statistically significant difference (two-way ANOVA with Tukey’s *post hoc* test. * *p* < 0.05; ** *p* < 0.01; *** *p* < 0.001; **** *p* < 0.0001). Genotypes of samples: Ctrl = Tubulin-Gal4/+; RNAi = Tubulin-Gal4/UAS D-idua^RNAi3^. STV indicates a starvation of 4 h.

We finally investigated the metabolic alterations associated with *D-idua* downregulation by analyzing the expression levels of genes involved in glycolysis/glyconeogenesis and lipogenesis, as these pathways are recognized to be altered in models with defective autophagy or lysosome dysfunction [54,55,56]. In Figure 8A, the genes used to evaluate glucose metabolism are marked in red: ATP-dependent 6-phosphofructokinase (Pfk), one of the main regulatory enzymes in glycolysis; triose phosphate isomerase (Tpi), essential for efficient ATP production; and lactate dehydrogenase (Ldh), which catalyzes the conversion of glycolysis-derived pyruvate to lactate. As shown in Figure 8B, expression of glycolytic enzymes was increased 3-fold in our *D-idua* model compared to control, suggesting the induction of glycolytic flux. As expected, starvation slightly decreased glycolytic gene levels in control larvae and totally restored the expression levels of *D-idua* glycolytic gene transcripts, in line with the cellular phenotypes. The lipogenic pathway is strictly connected to glycolysis trough the citrate derived from the TCA cycle that provides the source of cytosolic AcCoA (Acetyl Coenzyme A). We registered a 5-fold increase of *Acetyl-CoA carboxylase* (*ACC*), encoding a ubiquitous metabolic enzyme catalyzing the carboxylation of acetyl-CoA to malonyl-CoA, the rate-limiting substrate for fatty acid synthesis (Figure 8C), supporting the idea that the loss of *D-idua* activates de novo lipogenesis in vivo.

Starvation greatly reduced the expression levels of *ACC*, *ACLs* and *FASN1* in control larvae and ameliorated D-idua^RNAi3^ lipogenic gene expression. Such data show that the induction of glycolysis in *D-idua* larvae is accompanied by an increased de novo lipogenesis, resulting in coordinated changes of metabolic gene expression.

## 4. Discussion

In this paper we presented the characterization of a novel *Drosophila* model of MPS I, showing that the fruit fly homologue of IDUA, D-idua, maintains a structural and functional conservation with the human counterpart. We showed that *D-idua* is a vital gene and that the reduction of its expression is associated with premature death and an age-dependent climbing decline. In addition, we observed dysfunctions of lysosomes (Figure 4 and Figure 5) and of the autophagic pathway (Figure 6 and Figure 7), together with metabolic changes in glycolysis and lipogenesis (Figure 8). Interestingly, starvation was shown to greatly ameliorate these altered phenotypes.

Lysosomal hydrolases are ubiquitously expressed, and their partial or total loss of activity can result in damage to different organs and tissues, leading to a wide range of clinical manifestations with premature death. In our specific case, ubiquitous reduction of D-idua activity in *Drosophila* caused developmental defects leading to premature death at different stages, depending on the tissue and on the extent of the expression reduction (Figure 3). Muscle-specific downregulation of *D-idua* led to death at pupal stage suggesting that *D-idua* is a key player in muscle development and/or maintenance. This possibly reminds of musculoskeletal difficulties, as well as the important cardiac involvement, typical of MPS I patients [57,58,59]. Our analysis further suggested a role of *D-idua* in CNS development and functionality. MPS I patients often present a significant CNS impairment, such as behavioral problems and cognition impairment [60]. Here we exclusively quantified negative geotaxis response; however, a more careful analysis of behavioral phenotypes could help to understand the role of *D-idua* in neurons and glia, as a possible measure of cognitive decline.

At the cellular level, our data demonstrated that the reduction of D-idua enzyme activity is associated with autophagy defects, in good agreement with what was previously observed in other LSD models [23,24,61]. In our fly model, lysosomes were inefficiently acidified in muscle tissue (Figure 5) and were unable to fuse to autophagosomes (Figure 6 and Figure 7), confirming what was previously seen in other MPS *Drosophila* and mouse models [18,24]. In the mucolipidosis type IV model, accumulation of autolysosomes and autophagosomes was observed, which identified a tight link between LSD pathogenic mechanisms and lysosomal/autophagy dysfunctions [23]. Muscle remodeling studies investigating the molecular basis of metamorphosis in *Drosophila* emphasized the role of lysosomes and autophagy during this process: it was shown that the expansion of a tubular autolysosomal network in muscles requires lysosomal function and the process depends on the autophagy conjugation system [62].

The exact mechanism inducing a block of autophagy and of autophagosome-lysosome fusion needs to be further investigated, although some hypotheses can be discussed based on the latest research. Pharmacological models of LSDs using bafilomycin A1 (BafA), a compound that inhibits vacuolar type H+-ATPase (v-ATPase) leading to alkalinization of lysosomes, showed autophagic flux dysfunction, characterized by a lack of autophagosome-lysosome fusion [63,64]. Moreover, BafA can up-regulate the transcription factor EB (TFEB), a substrate of mTORC1 that drives the transcription of many lysosomal and autophagy-related genes [64]. Starvation is a positive stimulus that induces autophagy and, via mTOR inactivation, activates TFEB and modulates cellular homeostasis [65]. The same mechanism could be possibly involved in the comprehension of the rescue phenotype we observed in the *D-idua* knockdown animals under the starvation condition.

Metabolic alterations are not well studied in lysosomal storage disorders, but metabolic shifts similar to those observed in our *D-idua* model have been previously observed in an MPS I mouse model, whose liver was deficient in simple sugars, nucleotides, and lipids [66]. In our work, we found that the mRNA expression of some genes involved in glycolysis and lipogenesis was upregulated, suggesting that the upregulation is a response of the cell to deregulated sugar and lipid homeostasis. On the other hand, the energetic state of the cell and mitochondrial function might be affected in our *D-idua* model. Mitochondrial recycling defects have been observed in LSD models, showing an accumulation of dysfunctional mitochondria with an altered morphology [67]. It is known that the mitochondrial energy crisis increases lactate production, decreases fatty acid β-oxidation, and activates the catabolism of branched-chain amino acids to provide acetyl-CoA for de novo lipid synthesis [68,69]. The increased glycolysis coupled to the increased lipogenesis seen in the *D-idua* model suggest that cells may undergo a metabolic reprogramming, providing an alternative way to maintain an energetic balance.

The lifespan extension observed in neuronal and glial *D-idua* downregulated flies was certainly unexpected and requires further and appropriate investigations. Poor information is available on this issue in other LSD fly models and a comparison is therefore difficult. Most studies were focused on null mutant phenotypes that presented reduced lifespan and locomotor disability, whereas a tissue/cell specific analysis was often absent [25].

The way different types of cells have the ability to upregulate the lysosomal-autophagy pathway and the alternative strategies to dispose of the storage material remain unclear.

## 5. Conclusions

In this work we generated and characterized a *Drosophila* model of MPS I that allowed us to highlight significant alterations of districts, such as the muscle or the CNS. Such alterations represent, in a reproducible way, those affecting the homologous human districts. The identification of *D-idua* as a vital gene for *Drosophila*, the increased number and size of lysosomes, and their reduced acidification have shown, since the beginning, the possibility to employ our *Drosophila* as a model for MPS I. The model, thus, represents a useful instrument to obtain further information on these aspects, as well as on others related to the alteration of the autophagosome-endosome fusion, the autophagy system, and the conditioning of the starvation on these processes.

The analysis of the neurological compartment, together with further behavioral studies, may lead to the comprehension of the molecular alterations progressively leading to CNS impairment in the severe forms of MPS I. Finally, the consequent pharmacological screening by the testing of therapeutic molecules, easily evaluable through experiments of phenotype rescuing, will be performed much faster in the fly model.

In conclusion, the data shown here assert the representativeness of the model, highlighting alterations of the important cellular and lysosomal processes already known for the human disease. Therefore, this model will be of interest for the conduction of significant future studies on MPS I pathogenesis and treatment.

## Figures and Tables

**Table 1:** *Drosophila* strains used in the project’s development.

Strain	Source	Code
Tubulin-Gal4	Bloomington Drosophila Stock Center	BL 5138
Actin-Gal4	Bloomington Drosophila Stock Center	BL 4414
Mef2-Gal4	Bloomington Drosophila Stock Center	BL 27390
Elav-Gal4	Bloomington Drosophila Stock Center	BL 458
Repo-Gal4	Bloomington Drosophila Stock Center	BL 7415
UAS-GFP-mcherry-Atg8a	Bloomington Drosophila Stock Center	BL 37749
UAS-mcherry-Atg8a	Bloomington Drosophila Stock Center	BL 37750
w^1118^	Bloomington Drosophila Stock Center	BL 5905
UAS-GFP-Lamp1	Kindly provided by Helmut Krämer (Department of Neuroscience, University of Texas, Dallas, TX)	
UAS-Idua^RNAi1^	Vienna Drosophila Resource Center	13244/GD
UAS-Idua^RNAi2^	Vienna Drosophila Resource Center	103771/KK
UAS-Idua^RNAi3^	Vienna Drosophila Resource Center	13245/GD

**Table 2:** *Drosophila* primer pairs used for the qRT- PCR analysis.

Gene	Primer Sequences
*Idua*	Fw: 5′-GCCCTTCGACTTAATCTTCGCC-3′ Rv: 5′-GATTGCCCATCCACTCCAGAAC-3′
*Rp49*	Fw: 5′-AGGCCCAAGATCGTGAAGAA-3′ Rv: 5′-TCGATACCCTTGGGCTTGC-3′
*Tpi*	Fw: 5′-GACTGGAAGAACGTGGTGGT-3′ Rv: 5′-CGTTGATGATGTCCACGAAC-3′
*Pfk*	Fw: 5′-CTGCAGCAGGATGTCTACCA-3′ Rv: 5′-GTCGATGTTCGCCTTGATCT-3′
*Ldh*	Fw: 5′-GTGTGACATCCGTGGTCAAG-3′ Rv: 5′-CTACGATCCGTGGCATCTTT-3′
*Acc*	Fw: 5′-TAACAACGGAGTCACCCACA-3′ Rv: 5′-CAGGTCACAACCGATGTACG-3′
*Fasn*	Fw: 5′-CGTACGACCCCTCTGTTGAT-3′ Rv: 5′-AGTGCAAGTTACCGGGAATG-3′
*Acly*	Fw: 5′-TCCGGCAAGGACATCCTGA-3′ Rv: 5′-GGAATTTACTGTGGAAAAACGGC-3′

## Data Availability

The datasets generated and/or analyzed during the current study are available from the corresponding author upon request.
